# Management of Complex Infections in Hemophagocytic Lymphohistiocytosis in Adults

**DOI:** 10.3390/microorganisms11071694

**Published:** 2023-06-29

**Authors:** Yi Zhang, Zhipeng Cheng, Yu Hu, Liang V. Tang

**Affiliations:** Institute of Hematology, Union Hospital, Tongji Medical College, Huazhong University of Science and Technology, No. 1277 Jiefang Avenue, Wuhan 430022, China

**Keywords:** hemophagocytic lymphohistiocytosis, complex infections, hematological malignancy, differential diagnosis, management

## Abstract

Hemophagocytic lymphohistiocytosis (HLH) is a syndrome of excessive immune system activation and inflammatory response due to a variety of primary and secondary factors that can cause a range of clinical symptoms and, in severe cases, life-threatening conditions. Patients with HLH are at increased risk of infection due to their abnormal immune function as well as chemotherapy and immunosuppressive therapy at the time of treatment. At the same time, the lack of specific clinical features makes complex infections in HLH challenging to diagnose and treat. The management of complex infections in HLH requires a multidisciplinary and integrated approach including the early identification of pathogens, the development of anti-infection protocols and regimens, and the elimination of potential infection factors. Especially in HLH patients with septic shock, empirical combination therapy against the most likely pathogens should be initiated, and appropriate anti-infective regimens should be determined based on immune status, site of infection, pathogens, and their drug resistance, with timely antibiotic adjustment by monitoring procalcitonin. In addition, anti-infection prophylaxis for HLH patients is needed to reduce the risk of infection such as prophylactic antibiotics and vaccinations. In conclusion, complex infection in HLH is a serious and challenging disease that requires vigilance, early identification, and timely anti-infective therapy.

## 1. Introduction

### 1.1. Definition and Classification of HLH

Hemophagocytic lymphohistiocytosis (HLH) is a rare but serious disease that can be life-threatening in severe cases. The pathogenesis of HLH is the over-activation of the immune system due to various causes, resulting in the excessive activation of cytotoxic T lymphocytes, natural killer (NK) cells, and macrophages and the release of cytokines to attack normal tissues, leading to tissue damage and organ failure [1]. HLH can occur in people of all ages [2], but is relatively rare in adults. A national study of pediatric and adult cases in Japan estimated the annual incidence of the disease to be 1:800,000 [3], while a Swedish study reported a 0.9% incidence of adult malignancy-associated HLH in oncology patients [4].

Under normal conditions, CTL and NK cells can release cytolytic granules containing perforin and granzyme to promote the lytic destruction of target cells. After the target cells are destroyed, the immune activation stimulus signal is eliminated and the immune response is effectively controlled. The orderly conduct of this process depends on the normal structure of perforin and granzyme, intracellular transport, packaging, exocytosis, and immune synapse formation [2]. When this process is abnormal, CTL and NK cells are unable to effectively clear antigenic stimuli and are thus continuously activated. The persistently activated cells release large amounts of cytokines such as IFN-γ, TNF-α, IL-6, IL-18, IL-1β, and other cytokines, resulting in a cytokine storm, which causes tissue and organ damage. The etiology of HLH includes primary and secondary causes. Primary HLH is usually driven by an underlying genetic mutation involved in this pathway. The main recognized primary HLH-related genes are familial HLH: PRF1, UNC13D, STX11, and STXBP2; X-linked lymphoproliferative disorder: SH2D1A, BIRC4; Griscelli syndrome type 2. RAB27A; Chediak–Higashi syndrome: LYST, etc. [1]. In contrast, secondary HLH is usually associated with infections, autoimmune diseases, malignancies, chimeric antigen receptor T-Cell immunotherapy (CAR-T therapy), etc. [5].

The clinical manifestations of HLH are nonspecific and include fever, anemia, hepatosplenomegaly, and central nervous system symptoms. Laboratory tests often show coagulation disorders, liver dysfunction, leukopenia, thrombocytopenia, hypertriglyceridemia, elevated ferritin, hemophagocytosis, and decreased NK cell activity [6].

Because HLH can lead to organ failure and is life-threatening in severe cases, early diagnosis and treatment are required. Current diagnosis and treatment are based on the HLH-2004 criteria originally developed for the pediatric population: the diagnosis of HLH is based on eight criteria (fever, splenomegaly, cytopenia of more than two lines, hypertriglyceridemia and/or hypofibrinogenemia, hemophagocytosis, hypo/deficient NK cell activity, elevated ferritin, and high sCD25) that must be met for five of the criteria, unless there is a clear family history of primary HLH or the molecular diagnosis is consistent with primary HLH. The specific criteria are shown in Table 1. Treatment of HLH in adults is mainly based on the HLH-1994 regimen, with the overall goal of inducing and maintaining a steady state with etoposide and dexamethasone, and ultimately, curing primary, persistent, and recurrent HLH by hematopoietic stem cell transplantation (HSCT). A prospective clinical study showed a 5-year survival rate of 66 ± 8% in patients receiving HSCT after the 1994 regimen [7]. However, the treatment of adult patients with HLH still needs to be adapted to the etiology and complications based on the 1994 regimen [8].

### 1.2. Definition and Classification of Complex Infections

There is no clear concept to define complicated infections, and we have yet to define complicated infections as those that occur in immunodeficient patients and present with complex types of pathogens and complex sites of infection. The pathogens of complex infections are usually multi-drug resistant strains and are characterized by high morbidity, high lethality, and high economic burden.

### 1.3. Reasons for the High Prevalence of Complex Infections in HLH

**Figure 1:**
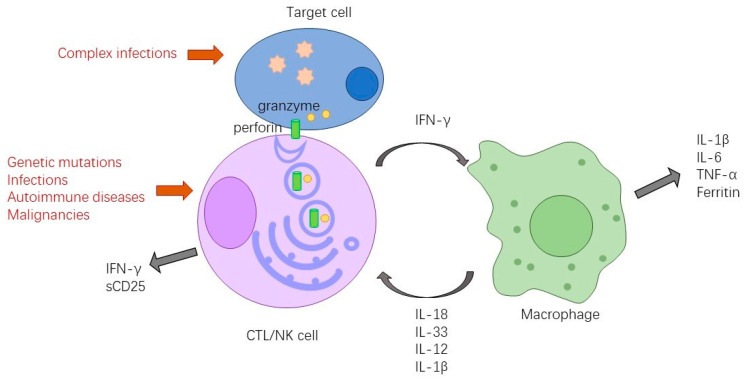
Pathophysiological processes of HLH.

The high prevalence of complex infections in patients with HLH is associated with multiple causes. First, HLH itself has abnormal immune function and its pathogenesis involves multiple immune and inflammatory mechanisms [9], as shown in Figure 1: earlier studies have shown that the vast majority of patients with HLH have largely intact humoral immunity but significantly impaired cellular immune function, while significantly reduced cytotoxic T cell toxicity and absent NK cell toxicity were found, suggesting that patients with active HLH may have complete cytotoxic dysfunction. This finding is related to the pathophysiological process of HLH disease itself, independent of the treatment [10]. Additionally, patients with HLH have an abnormal cytokine status due to a pathological inflammatory response such as high levels of tumor necrosis factor-α (TNF-α) and interferon γ (IFN-γ), which can lead to myelosuppression and thus cytopenia [11]. Currently, a cohort study of children reported that 10 out of 18 (56%) children with primary HLH developed severe infections during treatment and 67% of deaths were directly attributable to invasive infections [12]. Recent studies have shown that infection can cause 39.4% of direct deaths when HLH is not the direct cause of death [13]. Second, HLH is often secondary to immune system diseases and malignant hematologic neoplasms, which themselves as well as the chemotherapy and immunosuppressive therapy they receive, can also lead to mucosal damage, immune dysfunction, and cytopenia, further increasing the likelihood of complex infections. A study of tissue necrotizing lymphadenitis-associated HLH (HNL-HLH) stated that 93.8% (15/16) of patients had leukopenia and/or neutropenia at the time of diagnosis of HNL-HLH [14]. Thus, complex infections are more prevalent in patients with HLH.

### 1.4. Dilemmas in the Diagnosis and Treatment of Complex Infections in HLH

HLH and complex infections are inextricably linked, and their diagnosis and treatment face several challenges. On one hand, infection is a common trigger of HLH, especially in intensive care units, where it is considered to be the most common trigger of HLH, accounting for 42.5% of all causes [15]. On the other hand, infection is also a common complication of HLH, although no studies have reported the incidence of infection in adult patients with HLH yet. In terms of diagnosis, there are some difficulties in the differential diagnosis between HLH and complex infections because of the crossover of clinical symptoms and laboratory tests between these two. For example, both HLH and sepsis may lead to fever, leukopenia, methemoglobinemia, and thrombocytopenia due to disseminated intravascular coagulation (DIC) [16]. In addition, there are several therapeutic paradoxes associated with complex infections in HLH. The treatment of complex infections is mainly based on antibiotics and symptomatic supportive therapy but does not include the key drugs for HLH treatment. In contrast, the treatment of HLH is mainly based on immunosuppressive therapy, which may lead to exacerbation of the infection, making the development of a treatment plan very difficult.

HLH is a rare and difficult-to-diagnose disease; therefore, there are fewer studies on complex infections in HLH and a lack of clinical data to develop effective strategies to prevent and treat the infection. The recommendations in this article are extrapolated from the patterns of complex infections in other hematologic diseases such as HSCT and febrile neutropenia. The purpose of this review is to summarize the current literature on complex infections in adult and pediatric patients with HLH and to make recommendations for the prevention and treatment of complex infections in HLH.

## 2. Current Status of Complex Infections in HLH

The current status of complex infections in HLH is unclear and there are fewer studies. In a clinical study, 12 (60%) of the 20 pediatric HLH patients died, with eight deaths attributed to invasive infections including disseminated cytomegalovirus infection (1/20), enterobacterial sepsis (1/20), and invasive fungal infections (6/20) [17]. A recent case report reported a 21-year-old male patient on HLH treatment who presented with proton pump inhibitor (PPI)-refractory gastritis-like symptoms and subsequent gastroscopy suggestive of gastric trichomycosis, after which the patient was treated with antifungal drugs and the necrotic gastric tissue was removed [18]. The paucity of studies of complex infections in HLH may be related to the fact that infection is a secondary cause of HLH, and current reports have focused on infection as a cause of secondary HLH. Although not well-studied, the available studies suggest a high incidence of complex infections and infection-related mortality in HLH.

## 3. Diagnosis and Differential Diagnosis of Complex Infections in HLH

### 3.1. Diagnosis of Complex Infections in HLH

Complex infections in HLH often occur at different sites, most commonly in the lung, soft tissue of the skin at the indwelling catheter, gastrointestinal tract, and urinary tract. Patients with fever during or after HLH treatment should be alerted to infection and clinically evaluated for infection, site of infection, and type of pathogen as early as possible for timely medical intervention.

When a patient with HLH has a fever or suspected infection, in addition to routine blood analysis, biochemical indicators such as C-reactive protein (CRP) and calcitoninogen (PCT) to assess the patient’s inflammatory status, tests such as pathogenic culture and antigen detection are performed on samples of the patient’s blood, urine, stool, anal swabs, sputum, bronchoscopic lavage, and other possible sources of infection to look for pathogens. It is important to note that pathogenic cultures should be performed when infection is suspected and before antimicrobial therapy, and usually include at least two groups: aerobic and anaerobic. Obtaining cultures before the administration of antibiotics can significantly increase the yield of cultures and thus make it more likely that pathogens will be identified [19]. In addition, intravenous catheters left in place to facilitate treatment in patients with HLH are a potential source of infection, and at least one set of blood cultures (along with peripheral blood cultures) should be obtained from the catheter in the event of unexplained fever or suspected catheter related blood stream infection (CRBSI). If a positive blood culture from the catheter is obtained 2 h before a positive peripheral blood culture, it indicates that CRBSI has occurred [20]. The aforementioned CRP is dependent on the release of inflammatory factors during infection, is elevated within 24 h of infection, and can predict the progression of infection [21], and PCT is specific for the diagnosis of bacterial infections [22]. In addition, new serological methods such as aspergillus galactomannan antigen and β-D-glucan assays are useful in the diagnosis of fungi [23]. Currently, with the development of sequencing technology, macro-genome next-generation sequencing (mNGS) and tri-generation sequencing based on the nanopore platform can diagnose pathogens more rapidly, providing new options for infected patients. A retrospective study showed that mNGS is more sensitive in peripheral blood samples from HSCT patients than traditional microbial assays and that mNGS takes less time to identify multiple microbial infections [24]. In addition, the use of nanopore-targeted sequencing (NTS) technology in HSCT patients has shown that the NTS-guided adaptation of anti-infection regimens improves anti-infection efficiency and reduces infection-related mortality in patients with HSCT [25]. In addition to the laboratory tests above-mentioned, imaging tests such as chest X-ray, abdominal computed tomography (CT), and magnetic resonance imaging (MRI) of the head can be used to identify the site of infection.

### 3.2. Differential Diagnosis of Complex Infections in HLH

A major challenge in the differential diagnosis of patients with fever during or after treatment for HLH is distinguishing between infection and HLH relapse. The 2019 publication Recommendations for the management of hemophagocytic lymphohistiocytosis in adults points to HLH secondary infections as a major cause of death, which may be misdiagnosed as HLH relapse (Strong Consensus) [26]. Complex infections have a similar high inflammatory state as HLH, and fever and leukopenia can be seen in both HLH and complex infections [27]. The treatment of complex infections is based on anti-infective and symptomatic supportive therapy and does not include key drugs for the treatment of HLH. In contrast, the treatment for HLH relapse includes chemoimmunotherapy such as etoposide and dexamethasone, which may lead to myelosuppression and exacerbate the infection to some extent [28]. Therefore, the principles of treatment differ between the two, and differential diagnosis of patients with suspected infection or recurrence of fever during or after HLH treatment is essential so that the best treatment decision can be made.

**Figure 2:**
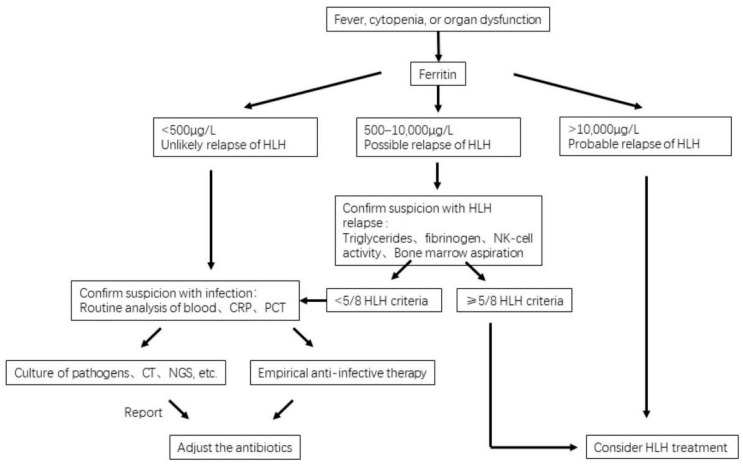
Differential diagnosis and management of complex infection and HLH relapse.

There is no single typical marker of HLH and therefore no single marker to distinguish between HLH relapse and complex infections. Studies are showing elevated ferritin (≥500 ng/mL), splenomegaly, marked peripheral cytopenia (hemoglobin <9 g/dL; platelets <100 × 10^9^/L; neutrophils <1 × 10^9^/L), hypofibrinogenemia (≤150 mg/dL), characteristic cytokine profile, and hypertriglyceridemia (≥3.0 mmol/L), supporting the diagnosis of HLH relapse [16]. The most important diagnostic marker in this regard is elevated ferritin. Although ferritin is associated with infection as an acute phase reactant, the literature shows that it is very helpful to differentiate between infection and HLH relapse when it is higher than 13,405 ng/mL [29]. Splenomegaly may be associated with a variety of abnormalities but is usually rare in patients with infections. In contrast, splenectomy is an important risk factor for infection, and common pathogens of splenectomy are *Streptococcus pneumoniae*, *Haemophilus influenzae*, and *Neisseria meningitidis* [30]. Therefore, splenomegaly contributes to the differential diagnosis of complicated infections and HLH relapse in the absence of other known causes. Although both leukocytosis and leukopenia may be present in infected patients, leukocytosis was predominant, with leukocytosis present in 51.2% of a study that included 5909 patients with suspected infection, while leukopenia was present in only 4.2% [31]. Thus, fever with significant leukopenia after HLH treatment needs to be considered as a possible HLH relapse. Fibrinogen is an acute-phase reactant whose concentration is usually elevated in patients with severe infections but may be decreased when DIC is present and is associated with increased mortality in intensive care unit (ICU) patients [32]. However, in HLH, fibrinogen activators released by macrophages can lead to a decrease in fibrinogen [33], so isolated hypofibrinogenemia can indicate HLH relapse, but when DIC is present, this criterion is of little clinical significance. In addition, triglycerides are often significantly elevated in patients with HLH because elevated TNF-α inhibits lipoprotein lipase [34], whereas triglycerides are not or only moderately elevated (>2.0 mmol/L) in infected patients [35], hence, elevated triglyceride levels can strengthen the suspicion of HLH in adults. Cytokine analysis is of high value in the differential diagnosis of infection and HLH recurrence, and researchers are trying to determine which biomarkers can be used as predictive tools. A prospective study that included 756 pediatric HLH patients found that a specific cytokine pattern with significantly elevated levels of IFN-γ and IL-10 and only moderately elevated levels of IL-6 had high diagnostic accuracy for HLH and could be used to differentiate HLH from infection [36]. It has also been shown that a predictive model consisting of IL-8, IL-1β, and IFN-γ has good sensitivity and specificity for identifying serious infections [37]. However, studies have also examined HLH-related cytokines such as IFN-γ, TNF-α, IL-6, IL-10, and IL-15 in patients with HLH associated with CAR-T therapy, but found no difference in the cytokine levels between infected and uninfected patients [38]. It is also noteworthy that hemophagocytosis is neither specific nor sensitive to HLH, and its presence does not exclude infection [39,40]. Decreased NK cell activity and elevated sCD25 levels can be seen in both HLH and infection. This review summarizes common indicators for the differential diagnosis of complex infection and HLH relapse, as shown in Table 2. Figure 2 provides the flow of their differential diagnosis and treatment.

## 4. Treatment of Complex Infection in HLH

### 4.1. Initial Treatment: Empirical Broad-Spectrum Antibiotic Therapy Is Recommended as Soon as Possible after Identification of the Infection; Narrow the Scope of Empirical Therapy Once the Pathogen Is Identified; and Select the Appropriate Dosage Based on Drug Metabolism and Pharmacokinetic Characteristics

Early and rapid dosing is central to achieving a beneficial effect against infection. In the setting of sepsis or septic shock, delayed first antibiotic administration is associated with increased in-hospital mortality, with a linear increase in the risk of death for each hour of delay [41]. Several studies have shown that the risk of acute kidney injury [42] and acute lung injury [43] increases with increasing delay. Additionally, delayed anti-infective therapy increases the risk of developing septic shock in Gram-negative bacteremia infections [44]. Therefore, the early recognition of infection and the early administration of antibiotics are needed for patients with HLH.

The choice of empirical antibiotics depends on the patient’s clinical signs and symptoms, past history and epidemiologic risk factors, possible sources of infection, and severity of disease [45]. The selection of empiric therapy for HLH patients with a suspected infection should take into account clinical symptoms, site of infection, place of infection (e.g., community, chronic care facility, hospital), common local pathogen susceptibility patterns, and drug resistance. Recent studies have shown that Gram-negative organisms are isolated more frequently in hospital-acquired infections compared to community-acquired infections (71% vs. 51%), most commonly *Enterobacteriaceae* (26%), *Pseudomonas* (16%), and *Bacillus immobilis* (11%) [46]. In addition to Gram-positive and Gram-negative bacteria commonly found in complex infections in hematologic, the incidence of fungal infections is now rapidly increasing [47]. In addition, although previous studies have shown that viral infections account for only 3.7% of all infections in sepsis, viral and fungal infections also require attention in patients with HLH because viruses are a common cause of secondary HLH, and patients with HLH are in a state of immune disorder and are vulnerable to viral infections, recurrence of latent viruses in the body, and secondary infections.

In patients with HLH, the initial empiric anti-infective regimen should be broad enough to cover most of the pathogens that may be responsible for the infection. Initial drugs should cover typical Gram-positive and Gram-negative pathogenic organisms, with drugs covering anaerobes when intra-abdominal or anaerobic infections are considered, along with empiric antifungal or antiviral therapy as appropriate [48]. Initial treatment also requires the consideration of bacterial resistance, and in the past few decades, the focus of treatment for resistant bacteria has been on resistant Gram-positive bacteria such as *Methicillin-resistant Staphylococcus aureus (MRSA)* and *Vancomycin-Resistant Enterococcus (VRE)*. Currently, the infection rates for these pathogens have stabilized or declined [49]. The main concerns surrounding antimicrobial resistance now focus on Gram-negative bacteria including *Extended-Spectrum β-lactamase (ESBLs)*, *Carbapenem-Resistant Enterobacteriaceae (CRE)*, *Multidrug-Resistant Pseudomonas Aeruginosa*, and *Carbapenem-Resistant Acinetobacter Baumannii*. For HLH patients considering multi-drug resistant Gram-negative bacillary infections, combination therapy against Gram-negative bacteria is recommended for initial empirical treatment, and studies have shown that aminoglycosides have broader coverage than fluoroquinolones as combination drugs for patients with these severe infections [50]. Similarly, vancomycin, teicoplanin, or other anti-MRSA drugs can be used when risk factors for MRSA are present [51]. Macrolides or fluoroquinolones can be combined when *Legionella* infection is considered [52]. HLH patients have risk factors for invasive *Candida* infections such as immunocompromise, indwelling intravenous catheters, use of broad-spectrum antibiotics, and blood transfusions [53]. When candidemia and other invasive *Candida* infections occur, initial therapy may be fluconazole or one of the three approved echinocandins (caspofungin, micafungin, and anidulafungin), with liposomal amphotericin B and voriconazole as secondary alternatives. Echinocandins or liposomal amphotericin B are recommended as initial therapy with neutropenia [54]. Recent studies have also shown that intravenous administration of echinocandin monotherapy is as effective as other antifungals (amphotericin B, itraconazole) in the treatment of systemic candidiasis in immunocompromised patients while avoiding serious side effects such as nephrotoxicity caused by amphotericin B [55]. However, HLH-related mortality associated with histoplasmosis in adults is high, and early antifungal treatment with liposomal amphotericin B is essential [56].

Early optimization of antimicrobial pharmacokinetics can improve the prognosis of patients with complex infections in HLH. Different antimicrobial drugs have different optimal blood concentrations and dosing intervals due to different pharmacokinetics and pharmacodynamics, and the early administration of appropriate doses is essential to improve patient outcomes. Several studies have shown that irregular anti-infective dosing is associated with poor patient prognosis. Failure to achieve peak concentrations at the first dose is associated with the treatment failure of aminoglycosides in Gram-negative pneumonia [57]. Insufficient early vancomycin trough concentrations relative to the pathogen’s minimum inhibitory concentration (MIC) are associated with mortality and anti-infection failure rates in severe MRSA infections [58]. Success in treating hospital-acquired pneumonia and other serious infections is associated with higher peak concentrations of fluoroquinolones [59]. For beta-lactams, good anti-infective therapy and prognosis are associated with a longer duration of plasma concentrations above the pathogenic MIC, and studies have shown that the continuous infusion of beta-lactam antibiotics in patients with severe sepsis reduces in-hospital mortality compared with intermittent administration [60]. Therefore, it is important to rationalize and regulate the use of antibiotics in patients with complex infections in HLH according to the pharmacokinetics.

### 4.2. Patients with Septic Shock or Neutropenia in HLH Should Be Treated Empirically with a Combination of the Most Likely Pathogens at the Time of the First Anti-Infective Dose

The incidence of infections with drug-resistant organisms is increasing worldwide, and septic shock or neutropenia in HLH usually requires multidrug combination therapy against the possible pathogens. A retrospective multicenter cohort study showed that combination therapy improves survival in patients with septic shock [61]. A randomized controlled trial showed that patients with neutrophil deficiency in leukemia who received combination therapy had better anti-infective treatment than those who received monotherapy [62]. A prospective study enrolling 383 patients found that empirical treatment with third-generation cephalosporins combined with macrolides or fluoroquinolones in pneumococcal bacteremia was associated with lower mortality compared to monotherapy with beta-lactams [63]. However, the above multicenter cohort study also suggests that combination therapy may increase mortality in low-risk patients without septic shock [61]. Furthermore, a randomized controlled trial in 2012 showed that the combination of meropenem and moxifloxacin did not reduce the incidence of organ failure compared to meropenem monotherapy in adult patients with severe sepsis, and did not improve the prognosis of patients [64]. Although the findings are somewhat controversial, initial combination therapy is recommended in patients with septic shock, neutropenia, and specific pathogenic infections (e.g., *pneumococcal* infections) in HLH [65].

### 4.3. Patients with Septic Shock or Neutropenia in HLH Should Emphasize Early Downgrading When Using Combination Therapy; the Course of Therapy Should Be Extended Appropriately According to Immune Status, Site of Infection, Pathogen Type, and Drug Resistance; Infection Status Can Be Assessed by Monitoring PCT

The misuse and overuse of antibiotics are harmful to both society and individual patients: for society, the overuse and misuse of antibiotics may lead to an increased global epidemic of antibiotic resistance [66]; for patients, it may lead to adverse effects such as acute kidney injury and *C. difficile* infection [67]. Studies have shown that step-down therapy for septic shock is a safe strategy with low mortality, and the implementation of this strategy is fully justified [68], in addition to the economic advantages of optimizing antibiotic therapy through antibiotic step-down [69]. Therefore, early downgrading should be emphasized when using combination therapy in septic shock or neutropenia in HLH. However, there is a lack of consensus on the downgrading criteria for combination therapy, and the early downgrading of antibiotics can be based on clinical manifestations such as the resolution of shock symptoms, the recovery of PCT, and the return of pathogenic results [70].

In patients with HLH, the duration of anti-infection needs to be evaluated based on various aspects such as the type and resistance of the infecting pathogen, the site of infection, and the immune status of the patient. Despite the presence of potential risk factors such as the accumulation of antibiotic toxicity, secondary infections, and pathogen resistance, the duration of antimicrobial therapy needs to be extended in patients with complex infections in HLH. These conditions include HLH combined with multi-drug resistant pathogen infections, *Staphylococcus aureus bacteremia* (especially MRSA) [71], *Clostridium difficile (CDI)*, *Candida* [72], unspecified foci of infection, etc. A recent study stated that extended treatment of *C. difficile* infection beyond standard duration is associated with lower CDI recurrence rates [73]. The duration of treatment for uncomplicated candidemia should be 14 days after the first negative blood culture and the resolution of all associated symptoms. The site of infection may also affect the duration of treatment, for example, larger abscesses and osteomyelitis have limited drug penetration and require longer treatment times [74]. Although the course of anti-infective therapy is relatively prolonged in HLH with neutropenia, empirical anti-infective therapy can be discontinued after 72 h of recovery from fever and infection-related clinical symptoms, regardless of their neutrophil count, which reduces unnecessary antibiotic use and is safe [75].

It is also recommended that patients with sepsis and septic shock in HLH be evaluated daily for timely discontinuation and a reduction in antibiotic use. PCT, as an inflammatory indicator, can help distinguish bacterial from non-bacterial infections and is commonly used as an aid in the diagnosis of acute infections and the monitoring of infection status, and can therefore assist in determining the anti-infective regimen. Various PCT-based algorithms have been used to guide antimicrobial therapy for severe infections and sepsis [76]. A large randomized trial of PCT monitoring in critically ill patients with suspected bacterial infections demonstrated that PCT guidance reduced treatment duration and antibiotic dosage, and that this reduction was associated with a significant improvement in mortality [77]. It is important to note that PCT and all other indicators of inflammation can only provide supportive and complementary data for clinical evaluation and cannot be relied upon alone to determine the initiation, adjustment, and cessation of anti-infection.

### 4.4. The Fundamental Aspect of Complex Infections in HLH Is to Eliminate the Source of Infection and, If Necessary, to Remove the Central Venous Catheter

The etiologic treatment of complex infections in HLH is to identify the site of infection and anti-infective treatment while determining whether that site is suitable for source control measures. The goal of source control is to eliminate the source of infection, control the ongoing source of infection, and restore anatomic structure and function [78]. Because source control interventions may lead to further complications such as bleeding, fistulas, or unintentional organ damage, the choice of the optimal source control approach must weigh the benefits and risks of a particular intervention, the risk of infection transfer, against the probability of successful source control [79]. In general, the least invasive and most effective source control options should be used, but open surgical interventions should be considered when other interventions fail to achieve therapeutic results. Strategies used to achieve control include the drainage of abscesses, debridement of infected necrotic tissue, removal of potentially infected devices, and the control of ongoing sources of microbial contamination [80]. A particular concern for complex infections in HLH is CRBSI, and in critically ill patients with proven CRBSI or local complications, it is recommended that the catheter be removed as soon as possible, regardless of the pathogen [81]. In addition, in patients with sepsis or septic shock in HLH using a tunneled catheter, removal of the tunneled catheter and insertion of a temporary, non-tunneled catheter is recommended. Long-term CRBSI associated with pathogens other than *Staphylococcus aureus*, *Pseudomonas aeruginosa*, *Bacillus* spp., *Micrococcus* spp., *Propionibacterium*, *fungi*, or *Mycobacterium* may be attempted without catheter removal using systemic antibiotics and antimicrobial blockade [82].

## 5. Prevention of Complex Infections in HLH

The Recommendations for the management of hemophagocytic lymphohistiocytosis in adults noted that extensive antimicrobial prophylaxis against *Pneumocystis carinii* and *fungi* is recommended while HLH is under treatment. As these patients are at high risk of fungal infections, they often require secondary prophylaxis. In addition, antiviral prophylaxis is also recommended due to severe T-cell depletion [26]. Meanwhile, the appropriate vaccination of HLH patients is recommended to prevent infection.

### 5.1. Prevention of Pneumocystis carinii Pneumonia

Pneumocystis carinii pneumonia (PCP) has previously been reported in pediatric patients with HLH as a first episode, and the use of trimethoprim-sulfamethoxazole (TMP-SMX) to prevent PCP at the time of chemotherapy in HLH patients is recommended [83]. Studies have shown that low doses of TMP-SMX have good treatment outcomes while reducing mortality as well as reducing the overall financial burden and improving patient compliance with daily treatment plans [84]. TMP-SMX administered 2–3 times a week is the dosage for the primary prevention of PCP in adult HLH patients. When TMP-SMX is poorly tolerated or contraindicated, drugs such as pentazocine, atovaquone, and aminophene can be chosen as alternative treatments [85].

### 5.2. Prevention of Fungal Infections

Immunocompromised patients are at high risk for invasive fungal infections (IFIs), especially those with hematologic malignancies receiving remission-inducing chemotherapy and those receiving HSCT. Antifungal prophylaxis with an oral triazole or intravenous echinocandins is currently recommended during remission-inducing chemotherapy for acute myeloid leukemia and myelodysplastic syndromes during the neutropenic period associated with chemotherapy [86]. The IDSA guidelines on fungal prophylaxis for hematologic malignancies recommend that a mold-active triazole is recommended when the population level risk of Aspergillosis is >6% [87]. It has been reported that 25% of a cohort of 71 patients with HLH in the ICU had disease complicated by invasive aspergillosis, which may be a complication of HLH [88], and therefore, IFI prophylaxis is recommended for patients with HLH receiving remission-inducing chemotherapy.

### 5.3. Prevention of Bacterial Infections

There is some controversy regarding bacterial prophylaxis in patients with hematologic diseases. In a study of early antibiotic prophylaxis in autologous HSCT recipients, neutrophil count-driven prophylactic antibiotic therapy was independently associated with a reduction in febrile events and mean antimicrobial days [89]. In 2007, the European Conference on Infections in Leukemia (ECIL) panel recommended the use of fluoroquinolones (FQ) for antimicrobial prophylaxis in high-risk neutropenic patients with an expected duration of neutropenia of more than 7 days [90]. Subsequent studies have suggested that FQ prophylaxis may reduce the incidence of bloodstream infections but not overall mortality [91], and that the decision to prophylactically administer FQ to patients with HLH should be weighed against its impact in terms of toxicity in individual centers and local ecological changes. In addition, antimicrobial prophylaxis against Gram-positive bacteria does not improve all-cause mortality and additional antibiotics can lead to adverse events such as nephrotoxicity [92]. As such, the prophylactic use of antibiotics targeting Gram-positive bacteria is not recommended in patients with HLH.

### 5.4. Prevention of Viral Infections

Patients with HLH have a complex relationship with viral infections. On one hand, viral infections such as Epstein–Barr virus (EBV)*,* are secondary risk factors for HLH, and on the other hand, patients with HLH are prone to the recurrence of viral infections during treatment, and viral infections are predictors of poor prognosis in HLH [93]. The guidelines recommend that herpes simplex virus seropositive patients receiving allogeneic HSCT or leukemia induction therapy should receive antiviral prophylaxis such as acyclovir [87]. Additionally, previous studies have shown that entecavir reduces the incidence of hepatitis B virus (HBV)-related hepatitis and HBV reactivation in patients with diffuse large B-cell lymphoma who are serologically positive for HBV surface antigen on R-CHOP chemotherapy [94]. Therefore, antiviral prophylaxis is likewise recommended for HLH patients receiving chemotherapy. In addition to herpes simplex virus and HBV prophylaxis, cytomegalovirus prophylaxis is mainly performed using sensitive laboratory monitoring to determine the presence of viral replication and the early initiation of antiviral therapy such as ganciclovir [95].

### 5.5. Vaccination

Patients with malignant hematologic diseases are at increased risk for infectious complications, and vaccination is an effective preventive strategy against many infections. Data on vaccination strategies for patients with HLH are lacking, the role of vaccination is unclear, and careful selection of the type and timing of vaccination is required. Although inactivated unconjugated vaccines may not induce sufficient antibody production in immunocompromised patients, live vaccines are contraindicated in immunocompromised patients [96]. Guidelines recommend annual quadrivalent inactivated influenza vaccination for all patients receiving chemotherapy for malignant hematologic neoplasms [87]. However, there is no conclusive evidence that vaccination reduces influenza-related mortality [97]. Several cases of HLH following the COVID-19 vaccine [98,99,100] and one case of inactivated influenza vaccine [101] have been reported worldwide, so the immunogenicity and safety of vaccination in this population are unknown, and further studies are needed to guide future recommendations.

## 6. Conclusions and Future Directions

HLH patients have a high incidence of complicated infections and infection-related mortality due to the presence of multiple infection-related risks such as abnormal autoimmune status, combination chemotherapy, hemocytopenia, and the use of immunosuppressive drugs. At the same time, complex infections in HLH are more difficult to distinguish from HLH relapse due to the presence of overlapping clinical symptoms and laboratory tests. In addition, because HLH patients are inherently immunodeficient, their complex infections are more difficult and risky to treat, making their effective management critical. By closely monitoring the patients’ conditions, early diagnosis, integrated use of antimicrobial drugs, and effective anti-infection prophylaxis, complex infections in patients with HLH can be effectively controlled with as few side effects as possible.

However, there is a paucity of clinical and basic research on complex infections in HLH. Therefore, more clinical studies are needed in the future such as clarifying the pathogenic spectrum of complex infections in HLH, which will in turn provide an in-depth exploration of this area to provide better support and guidance for clinical practice. At the same time, there is also a need to strengthen the basic research on HLH and complex infections to gain insight into their pathophysiological mechanisms and to provide a basis for the development of more effective treatment plans.

## Figures and Tables

**Table 1:** Diagnosis guidelines used in the HLH-2004 trail [6].

The Diagnosis of HLH Can Be Established If Either A or B Is Fulfilled
(A) Molecular diagnosis consistent with HLH:
Pathological mutations of PRF1, UNC13D, STXBP1, RAB27A, STX11, SH2D1A, or XIAP
(B) 5/8 diagnosis criteria for HLH below:
Clinical symptoms
Fever (temperature > 38.5 °C for >7 days)
2. Splenomegaly (spleen tip palpated >3 cm below left costal margin)
Laboratory findings
3. Cytopenia involving ≥2 lineages (hemoglobin <9 g/dL; platelet count <100 × 10^9^/L; neutrophil count <1 × 10^9^/L)
4. Hypertriglyceridemia and/or hypofibrinogenemia (fasting triglycerides ≥3 mmol/L; fibrinogen ≤150 mg/dL)
5. Low or absent NK-cell activity
6. Ferritin ≥500 μg/L
7. sCD25 (sIL2Rα) ≥ 2400 U/mL
Histological examination
8. Hemophagocytosis in bone marrow, spleen, lymph nodes, or liver

**Table 2:** Differential diagnosis of complex infection and HLH relapse.

	Complex Infection	HLH Relapse
Similarities		
	Fever	
	Hemophagocytosis	
	Low or absent NK cell activity	
	Elevated sCD25	
Differences		
Molecular diagnosis	Absent	Present
Splenomegaly	Rare	Present
Ferritin	↑	↑↑↑
Leukocyte	↑↑↑/↓	↓↓↓
Fibrinogen	↑	↓↓↓
Triglycerides	↑↑/-	↑↑↑
CRP	↑↑↑	↑
PCT	↑↑	-
IL-10	↑	↑↑↑
IL-6	↑↑↑	↑
IFN-γ	-	↑↑↑

-, Normal; ↑, mildly elevated; ↑↑, moderately elevated; ↑↑↑, significantly elevated.

## Data Availability

No data have been generated for this article; all data were cited from the published literature.
